# Parent carer and disabled young people’s perspectives on the impacts of changes to service provision for children and young people in England during the COVID-19 pandemic: a qualitative study

**DOI:** 10.1136/bmjopen-2024-085144

**Published:** 2024-11-27

**Authors:** Hannah Merrick, Helen Driver, Lily Potts, Catherine Exley, Amanda Allard, Christopher Morris, Jeremy R Parr, Lindsay Pennington, on behalf of Resetting Services Group, Kulwinder Bola

**Affiliations:** 1 Population Health Sciences Institute, Newcastle University, Newcastle upon Tyne, UK; 2 Newcastle University School of Psychology, Newcastle upon Tyne, UK; 3 Council for Disabled Children, National Children's Bureau, London, UK; 4 Peninsula Childhood Disability Research Unit (PenCRU), University of Exeter Medical School, University of Exeter, Exeter, UK; 5 Cumbria Northumberland Tyne and Wear NHS Foundation Trust, Newcastle upon Tyne, UK; 6 Great North Children's Hospital, Newcastle upon Tyne Hospitals NHS Foundation Trust, Newcastle upon Tyne, UK

**Keywords:** COVID-19, developmental neurology & neurodisability, health services, caregivers

## Abstract

**Abstract:**

**Objectives:**

In England, the delivery of health, education and social care services changed substantially during COVID-19. Some services closed, some had reduced capacity and there was a shift to the use of telehealth. This study aimed to understand how families of children and young people with neurodisability experienced these service changes, what did or did not work well for them and what impact the service changes had on them.

**Design:**

Qualitative study with parent carers of children (aged 0–19 years) with neurodisability accessing children’s health, social care and education-based services during the COVID-19 pandemic.

**Setting:**

Participants were recruited from five local authority areas in England and interviewed via telephone and/or video call.

**Participants:**

48 parent carers (45 mothers, three fathers) were interviewed and nine young people (aged 8–16 years). Across the parent carers there were 55 children with neurodisability (43 males, 12 females), ranging from 3 to 19 years. Children had a range of diagnoses, including autism, attention deficit hyperactivity disorder, cerebral palsy, genetic conditions and epilepsy. Nine young people (aged 8–16 years; eight males, one female) were interviewed; two individually, three in a focus group and four with their parent carer.

**Results:**

Four themes were identified: (1) communication of service changes, (2) access to services during the pandemic, (3) impacts of service changes and (4) learning for future emergencies and resetting services. Communication of service changes was reported as poor and confusing. Access to services during the pandemic varied. Medical services continued with least disruption; therapeutic, education-based and social care services were severely disrupted. Service changes had a detrimental impact on families coping with high levels of medical care and physical and behavioural support. Young people experienced negative impacts of service change on their physical, mental and behavioural health.

**Conclusions:**

Services for children with neurodisability require a person-led, family centred approach with strong multidisciplinary team working. Findings indicated the need for improved communication within and between services, and between services and families and young people. Planning for future emergencies needs to factor in the specific health and care needs of children with neurodisability and maintain access to services, in particular, those accessed through schools.

**STRENGTHS AND LIMITATIONS OF THIS STUDY**

- Direct reporting from parent carers and young people provides their lived experience of disruption to education, social care and health services during the pandemic.
- Recruiting young people to take part in remote interviews was challenging, resulting in low participation numbers.
- Reporting across health, social care and education sectors allows broad understanding of impact of service changes experienced by families.
- Recruiting from five diverse local authority areas provided the opportunity to learn from a wide range of changes and experiences.

## Introduction

 Globally, services to children with neurodisability were subject to closure or severe disruption as measures and restrictions were put in place to limit the spread of COVID-19. Legislation, including a national lockdown implemented in March 2020 in the UK, aimed to reduce the spread of the virus.^1^ Alongside this, specific clinically vulnerable groups, including some children with neurodisability, were given additional instructions to ‘shield’ (ie, avoid contact with anyone outside their home). Many health and social care services that were deemed ‘non-urgent’ or ‘non-essential’ had to close or ran at reduced capacities in the initial stages of the pandemic. Following this, many services responded by moving swiftly to telehealth (phone and video calls) to deliver essential care to their service users, whereas in other services the response was often more erratic and inconsistent. Schools also closed, except for the children of keyworkers and vulnerable children; for example, children with an education and healthcare plan (EHCP), with a child protection plan, receiving social care services or who would have difficulty accessing remote education.^2^ Some special education services reported a lack of contact with external services, which left gaps in access to health and social care.^3^ Children with neurodisability (eg, epilepsy, autism, cerebral palsy) depend on services and education settings for medical, physical, social and behavioural intervention to manage their conditions and, in many cases, to provide support to them and their families.

The onset of COVID-19 and the associated restrictions were challenging to society but are known to have had a profound and severe impact on families with disabled children.^4^ Evidence indicates that children with neurodisability and their families are more vulnerable to increased poverty, isolation and mental health difficulties,^5^,^7^ all magnifying disproportionate impacts of the pandemic on them. The imposed restrictions negatively impacted social, emotional, physical and developmental health for children and young people with special educational needs and disabilities (SEND) in the UK.^8^,^10^ Children with neurodisability have experienced deterioration in their condition and increases in pain and behaviours that challenge,^11^ and continue to face extensive waits for assessment, healthcare, access to equipment and support to manage their conditions.

Of 4000 families responding to a survey by the Disabled Children’s Partnership, 76% reported the vital healthcare and support they relied on had stopped altogether, leaving family members to deliver all care and support.^12^ Parent carers have described how COVID-19 exacerbated the pre-existing lack of support provided to their families.^13^ Parent carers reported high levels of physical and mental health problems and exhaustion through the pandemic.^14 15^ Similarly, siblings have experienced increased anxiety and isolation as a result of the experiences during the pandemic.^12 16^

The Resetting Services to Disabled Children research aimed to learn from the pandemic to understand how services could be delivered better to provide high-quality care to children with neurodisability and their families in times of emergency and as the UK National Health Service is remodelled. This paper reports on interviews carried out with parent carers and children and young people with neurodisability to understand how they experienced service changes during the COVID-19 pandemic, what did or did not work well for them and what impact the service changes had on them. A parallel paper provides the perspectives of health, education and social care professionals supporting families.^17^

## Methods

### Participants and recruitment

Qualitative interviews were conducted with parent carers of children and young people with neurodisability between March 2022 and August 2022. Participants were recruited from five local authority areas in England, purposefully selected to be diverse based on geographical (urban/rural) and demographic (area deprivation) characteristics, and the number of organisations providing statutory services to disabled children. The study aimed to recruit up to 20 parent carers of children, aged 0–19 years with neurodisability and up to 20 children and young people with neurodisability from each area. Parent carer and child/young person participants could be from the same family or different families. Neurodisability was defined as

a group of congenital or acquired long-term conditions that are attributed to impairment of the brain and/or neuromuscular system and create functional limitations. A specific diagnosis may not be identified. Conditions may vary over time, occur alone or in combination and include a broad range of severity and complexity. The impact may include difficulties with movement, cognition, hearing and vision, communication, emotion and behaviour.^18^

Purposive sampling was used to ensure that a range of experiences were captured. Our sampling frame aimed to take into account child factors (age and severity of impairment in communication, mobility and behaviour) and family factors (household set-up and geographic locations).

Recruitment continued until we had sufficient data to answer our questions and ensure a broad range of views and experiences had been included. Information about the study was cascaded through local parent carer organisations (eg, through face-to-face discussion and emails) and through local ambassadors in each site who worked with disabled children. Local ambassadors shared the project with colleagues and families they work with and displayed a study advert poster in clinical settings. Researchers contacted organisations and charities in each local area supporting disabled children to share recruitment information via social media platforms (eg, Facebook and Twitter). Interested parent carers and young people registered via a link to an OnlineSurvey on the study webpage or by directly emailing the research team. The registration forms asked participants to provide the age of their child, their child’s category of need, which region they lived in, which parts of the study they were interested in and their contact details. These details were used to identify eligible participants in each of the five areas that were interested in taking part in an interview. Due to the number of registrations with interest to take part in an interview, all those that selected an interest within the five areas were contacted to take part. They were then contacted by email and provided with an information sheet. All participants documented consent prior to interview with an electronic or type-written signature on the consent form.

### Interview procedures

Semi-structured interviews with parent carers were conducted by videoconferencing (via Teams/Zoom) or by telephone. At the start of the interview, participants reconfirmed verbally consent to the recording of the interview, participation in the study and understanding that transcripts will be anonymised for analysis to keep participants’ identities and others mentioned in interviews confidential. The interview topic guide (see online supplemental file 1) included initial questions asking participants to describe the services they accessed prior to the pandemic, and how often they accessed them. This was used to then understand how each of these services changed during the pandemic with open-ended questions focusing on how and in what ways services changed for them during the pandemic, the impact of these changes on families, what worked well and did not work well and what would have made substantive difference to improve the experience of children, parent carers and families during the pandemic. Most the young people were interviewed via video call with their parent carer. One small focus group was held with a group of young people in person at their college (for interview topic guide, see online supplemental file 2). All interviews were transcribed verbatim during the interview using Microsoft Stream for Teams or Zoom. Transcripts were checked for accuracy, corrected and anonymised, removing any identifying information about the family and child, and specifically named services and places.

### Analysis

Data collection and analysis was iterative, following principles of the constant comparison method, a core component of grounded theory.^19 20^ This iterative approach allowed us to capture quickly emergent themes directly from the data, systematically look at who is saying what in which contexts and explore those themes and comparisons with the research team and our public and patient involvement (PPI) advisors.^21^ Three researchers (HM, HD, LPo) analysed the transcripts. Analysis was assisted by the Framework approach.^22^ An analytical framework was developed through open coding of initial transcripts and with reference to the EPOC taxonomy^23^ for service change codes. Data were charted using the framework matrix and then summarised by category.

### Reflexivity

The authors acknowledge the need for reflexivity in the research process. Analysis and interpretation were led by coders HM and HD and supported by LPe, CE and the wider research team. HM has no lived experience of neurodisability or having a family member with neurodisability. HD is a parent carer of a young person with neurodisability but not situated in any of the local authority areas included in this study. Researchers were conscientious in reporting the narrative of participants engaging in reflection and discussion to maintain objectivity. Coding and framework analysis has been shared with the wider research team drawing in clinical and qualitative perspectives. It is recognised that both HD and HM who led on recruitment, interviews and analysis are both white, female and university educated, which may have impacted on who responded to the research invitation and our perspectives and interactions. To mitigate the potential biases introduced by our positionality, we have also strived to maintain transparency in reporting our methods and findings. PPI engagement with stakeholders (parent carers and children and young people) from areas beyond recruitment and diverse backgrounds provided valuable feedback and critical appraisal throughout the research process, including the findings from interviews. The varied experience and diverse positioning of the research team and PPI engagement established trustworthiness throughout the research process.^24^

### Public and patient involvement and engagement

Public and patient involvement and engagement informed each stage of the study. Research design involved collaboration with parent carers, children and young people with neurodisability and representatives of advocate organisations: Parent Carer Forum, Peninsula Childhood Disability Research UnitPeninsula Childhood Research and the Council for Disabled Children Council for Disabled Children(table 1).

**Table 1:** Patient and public involvement and engagement activity

Section and topic	Item
1. Aim	The aim of PPIE was to ensure relevance and reliability of this research study. Children and young people with neurodisability and their parent carers provided a unique perspective on the changes to their services and lived experience of the impact of these changes.
2. Methods	Three groups were involved: Peninsula Childhood Disability Research UnitPeninsula Childhood Disability Research Unit Family Faculty Parent Carer Group. A project-specific parent carer advisory groups were recruited through parent advocacy organisations. A young person advisory group was recruited through specialist schools local to the researchers’ university. Each group met regularly at each stage of the research to review methodologies, recruitment processes and to review findings and our interpretation. Parent carer advisory groups met four times, and young people’s groups met four times. The advisory groups were initially asked to provide feedback on the interview tools. Later, findings from the interviews and interpretations were presented to the groups and discussed. Meeting materials were adapted to enable access for all group members including those with communication support needs. In the final meetings for each group, the interview findings were shared and discussed.
3. Study results	The advisory group helped with updating study information sheets and in particular advised on recruitment strategies for the young people and parent carers. The emerging themes were presented to both the parent carer and young people advisory groups to provide validation and additional context to the interpretation of the final interview findings.
4. Discussion and conclusions	PPIE confirmed the impact of service changes on families. This contribution directly informed the study and recommendations developed in the next stage of the research.
5. Reflections/Critical perspective	Young people contributing to the PPI suggested further opportunities to engage with the process. This included providing an accessible overview of the study structure and timeline, with clear visual identification and mapping of the PPIE process. Young people’s PPI engagement gave insight into some of the challenges they may face when participating in interviews, insights that could be factored into the project design at an earlier stage.

PPIEpublic and patient involvement and engagement

## Results

Forty-eight parent carers (45 mothers and three fathers) were interviewed between April and September 2022 (table 2). The parent carer families included 55 children with neurodisability (43 males and 12 females), ranging from age 3 to 19 years. Their children had a range of diagnoses, including autism, attention deficit hyperactivity disorder, cerebral palsy, genetic conditions and epilepsy. Twenty-four children attended mainstream education, 21 attended a specialist provision school and 10 were in education other than at school. Thirty-eight (69%) had been given a formal diagnosis before the COVID-19 pandemic, while 15 children were diagnosed/assessed during the lockdowns and restrictions in 2020 and 2021, and two children were still waiting for assessment at the time of interview. Nine young people (aged 8–16 years; eight males, one female) were interviewed: two individually, three in a focus group and four with their parent carer (table 2).

**Table 2:** Description of families interviewed

	Total	Area 1	Area 2	Area 3	Area 4	Area 5
	N (%)	N (%)	N (%)	N (%)	N (%)	N (%)
**Parent carers interviewed**	**N=48**	**N=12**	**N=3**	**N=16**	**N=13**	**N=4**
Gender of parent carers						
Female	45 (93.8%)	11 (91.7%)	3 (100%)	16 (100%)	11 (84.6%)	4 (100%)
Male	3 (6.2%)	1 (8.3%)	0	0	2 (15.5%)	0
Number of children with a neurodisability in family						
1	41 (85.4%)	10 (83.3%)	3 (100%)	12 (75%)	13 (100%)	3 (75%)
2	5 (10.4%)	2 (16.7%)	0	2 (12.5%)	0	1 (25%)
3	2 (4.2%)	0	0	2 (12.5%)	0	0
Gender of child with neurodisability (n=55)						
Male	43 (78%)	11 (78.6%)	2 (66.6%)	17 (77.3%)	10 (90.9%)	3 (60%)
Female	12 (22%)	3 (21.4%)	1 (33.4%)	5 (22.7%)	1 (9.1%)	2 (40%)
Age of child(ren) (years) (n=55)						
3–5	7 (13%)	0	0	2 (9.1%)	4 (36.4%)	1 (20%)
6–9	14 (26%)	9 (64.3%)	0	5 (22.7%)	0	0
10–13	16 (29%)	2 (14.3%)	1 (33.3%	9 (40.9%)	4 (36.4%)	0
14–16	10 (18%)	1 (7.1%)	1 (33.3%	3 (13.6%)	2 (18.2%)	3 (60%)
17–19	8 (14%)	2 (14.3%)	1 (33.3%	3 (13.6%)	1 (9%)	1 (20%)
Category of need*						
Specific learning difficulties	10 (18.2%)	2 (14.3%)	1 (33.3%)	6 (27.3%)	0	1 (20%)
Speech, language and communication needs	25 (45.5%)	6 (42.9%)	2 (66.6%)	10 (45.5%)	5 (45.5%)	2 (40%)
Hearing impairment	4 (7.3%)	2 (14.3%)	1 (33.3%)	1 (4.5%)	0	0
Multisensory impairment	3 (5.5%)	2 (14.3%)	0	0	0	1 (20%)
Severe learning difficulties	5 (9.1%)	2 (14.3%)	0	3 (13.6%)	0	0
Autism	39 (70.9%)	8 (57.1%)	2 (66.6%)	16 (72.7%)	10 (91%)	3 (60%)
Physical disability	15 (27.3%)	6 (42.9%)	2 (66.6%)	3 (13.6%)	3 (27.3%)	1 (20%)
Socio, emotional and mental health	20 (36.4%)	7 (50%)	0	10 (45.5%)	1 (9%)	2 (40%)
Visual impairment	4 (7.3%)	2 (14.3%)	0	0	1 (9%)	1 (20%)
Moderate learning difficulties	8 (14.5%)	0	1 (33.3%)	4 (18.2%)	3 (27.3%)	0
Profound and multiple learning difficulties	5 (9.1%)	2 (14.3%)	1 (33.3%)	1 (4.5%)	0	1 (20%)
**Youngpeople interviewed**	**N=9**	**N=2**	**N=1**	**–**	**N=6**	**–**
Gender						
Male	8 (88.9%)	2 (100%)	1 (100%)	–	5 (83.3%)	–
Female	1 (11.1%)	0	0	–	1 (16.7%)	–
Age (years)						
6–9	1 (11.1%)	1 (50%)	0	–	0	–
10–13	1 (11.1%)	0	1 (100%)	–	0	–
14–16	7 (77.8%)	1 (50%)	0	–	6 (100%)	–
Category of need						
Autism	8 (89%)	2 (100%)	1 (100%)	–	5 (83%)	–
Hearing impairment	2 (22%)	–	1 (100%)	–		–
Physical disability	2 (22%)	–	–	–	2 (33%)	–
Moderate learning difficulties	2 (22%)	–	–	–	2 (33%)	–
Speech, language and communication needs	2 (22%)	–	–	–	2 (33%)	–
Socio, emotional and mental health	2 (22%)	–	–	–	1 (17%)	–

* Many children and young people had more than one diagnosis, therefore percentage adds up to more than >100%. Categories of need are from those used in the SEND Code of Practice in the UK.

The analysis established four overarching themes: (1) communication of service changes during the pandemic, (2) access to services during the pandemic, (3) impacts of service change and (4) learning for future emergencies and resetting services.

### Communication of service changes during the pandemic

Communication was reported as poor and confusing across services and sectors. Parent carers described challenges in being able to find out if services were available. They did not know who to contact for clarity, advice or information.

felt like the phone lines had been cut (Young person, A4_09)

I mean like even though the services have stopped like such as [hospital] and school, you’d have thought that they’d have something that they need to contact people, wouldn’t you? I mean, I know people working from home and stuff. I mean, I know there’s a massive wait list and stuff. But if you were due review, really, I should have got a phone call, shouldn’t I? Or a text? (Parent carer, A3_14)

Some parent carers had received letters advising them to shield their child. These instructions were given to people who were deemed clinically vulnerable and required them to stay at home, minimising contact with people outside their immediate household. Others had not received such instructions despite expecting they would. Some parent carers described not being able to get a consistent message from service providers about the vulnerability of their child to COVID-19 and the need to shield.

And then the school was saying he needs to be shielding, you know, because of his cerebral palsy, which was one of the shielding conditions. But the [paediatrician] was saying, no, don’t, even if it’s affecting their lungs and their chest, which it doesn’t. That was conflicting so [child] didn't know if he was coming or going. (Parent carer, A5_04)

Where there was communication about shielding and services, some messages caused anxiety; it was difficult to get agreement on shielding for children or get transparency on criteria for access to services. Uncertainty around the impact of COVID-19 on disabled children and young people led to fear and anxiety about accessing diagnostic, intervention and other services, in particular going into hospitals. Parent carers also referred to reading and hearing about ‘do not resuscitate’ plans at the beginning of the pandemic and their fear that their child would not be resuscitated or given necessary life support if they became severely ill with COVID-19.

I thought, if my son gets COVID, they’re going to let him die. They’re not gonna put him on life support. And I was right. We’ve seen what’s happened, learning disability six times more likely… But I was well aware they would not put him on life support if it was a choice between him and a family man with two kids. (Parent carer, A2_01).

Parent carers reported closure or stopping of multiple services, including those providing short breaks (ie, residential centres, day care services, overnight breaks), school services, equipment services and allied health therapies in the initial stages of the pandemic. The rationale for decisions on closures in the first lockdown was seen as unclear and led parents to question the criteria on which they were taken. Parent carers particularly questioned whether the impact of changes to children’s services on the outcomes for children and young people with neurodisability had been fully considered in decision-making processes.

In situations like [child]’s, on an individual, or at least the group basis, a lot of the policies might be appropriate for the general population, they provide the bulk protection, they provide some benefit to the individuals involved and they have relatively little costs, but the same actions have huge costs for (Son). So, here a different policy is appropriate. So, minimize expected harm in appropriately differentiated terms. So, to properly value the negative consequences too. (Parent carer, A1_02)

Many parent carers reported having very little contact from the services they usually accessed, including medical, allied health and short break services, other than to cancel appointments and notification that services would be in touch when appointments were available again, which lead to uncertainty. There was an overriding narrative of not being able to call services and not knowing how to access support or information. Some parent carers described losing contact with medical services, therapies, respite services and personal assistant care staff for extended periods of time throughout the pandemic.

And then about six months in, we had a video call from SALT [speech and language therapist], but maybe about nine months in, they kind of disappeared, any health service really disappeared like I guess what they classed as non-essential. I think most of the health workers I suppose like speech and language therapists and the physios there were drafted to work in COVID wards and things like that. So they completely disappeared for quite a long time. (Parent carer, A4_14)

Parent carers felt that school leadership staff faced pressure from lack of definite guidance and too much autonomy, leaving them to navigate the decision making for their school whereas school leaders should have been given clear, firm guidance. Parent carers described the burden of trying to make the right decisions for their children on whether to send them to school or not, and whether to access in-person services without having a clear understanding of COVID-19 or how to keep their child physically and mentally healthy.

it felt like they [school] were able to just operate as they wanted to, just they were given so much autonomy, which I felt wasn’t fair on them or us, because actually what it did was put the pressure on them, that if they were given the autonomy and made the wrong decision and goodness knows a vulnerable child caught COVID and was very ill with it, or worst case scenario died, then that was leaving that pressure on the head teachers. And I felt that the school should have been given clear firm guidance, and not been given, not had the pressure of the autonomy just been told what to do and yeah and, it was, that was the biggest blow to us. (Parent carer, A1_03)

### Access to services during the pandemic

The first change most parent carers described was the closure of schools or the restrictions placed on accessing schools, and the loss of school-based therapy.

School shut straight away. I’m in like the support bit of the college, like additional learning support at the college for various reasons. And we got told that the whole college would be closed for COVID. But we were told we would only be closed for a few weeks and then we’d be going back in…. And then that turned into weeks and then that turned into months and then that turned into a year and then that turned into 2 years. (Young person, A4_005)

The majority of families reported their child’s school closed for at least some period of time during the pandemic. Despite having an EHCP, which stipulates the care and support disabled children must receive, many parent carers said their children were not able to attend their school due to closure, or limitations on the number of places available. Families described how they lost access to education support and school nurses, as well as access to therapy services provided in education settings, such as physiotherapy, speech and language therapy and other specialist services such as hydrotherapy. For many children and young people, therapy was embedded in their curriculum and delivered through learning support or teaching assistants, this too was no longer delivered due to restricted school provision and remote or no access to school. Parent carers discussed challenges they had faced in getting an EHCP for their child before the pandemic, and how such challenges were amplified. Parent carers questioned the processes that were being used to monitor what schools were delivering during this time. For parent carers of children with visual and hearing impairments, the closure of schools meant access to specialist supports was lost, with little to no support provided for home-schooling. For parent carers of autistic children, the loss of school hugely disrupted routines and access to people and environments that supported their child. There were mixed opinions across the parent carers about remote support from schools while their children were at home. Some reported having no contact from school staff for long periods during the pandemic, while others reported receiving phone calls from school staff to check in, receiving helpful packages in the post containing resources and materials to support their child’s learning while they were away from school.

It was us that had to just chase school. They weren’t very good at keeping in touch, and they’d change times of things… with [Child] he is very routine led so we had done him like a timetable and then they send out messages quite frequently, you know the technical problems, the zoom meeting will now be at 10am not 9am. And [Child] will have got up at seven to anticipate in that nine o’clock meeting and then you’d have to find something else do with him to occupy until the 10 o'clock one. (Parent carer, A3_02)

The support he got from primary school was very good and obviously primary school he had a one-to-one support who we kept in touch with throughout. His class teacher would set tasks for him to do every day. (Parent carer, A4_16)

There was substantial disruption to short breaks provision, as well as to personal assistants and carers being able to visit homes. As a result, parent carers described the need to provide 24/7 care and support to their children. Parent carers reported how short break services were closed for extended periods and some had not fully reopened at the time of interview, 2 years after the first lockdown. Where short break services had begun to reopen, there was no offer of additional hours to compensate families for lost support. Additionally, where capacity was reduced due to ongoing restrictions (fewer or shorter sessions), families were not provided with alternative short break provision.

It stopped. So [respite centre] …, that just stopped, and I was told that she won’t be having any respite until further notice. And no additional hours were put into place or respite was put into place. (Parent carer, A5_02)

Parent carers described that many therapy services that stopped at the beginning of the pandemic were slow to restart, or never restarted, leaving children and young people without therapeutic care for prolonged periods; the impact on their physical health is described in theme 3 below. Many parent carers reported that, at the time of interview, some services had not yet returned to prepandemic levels of provision, and that young people were still experiencing difficulty accessing therapy and intervention support services. Longer delays accessing services and for assessments during the pandemic were widely reported by parent carers. Parent carers described continuing long waitlists for assessments, diagnostic, allied health, mental health and social services. They knew from personal experience, peers and feedback from service providers that these waits were not unusual before the pandemic but were exacerbated by COVID-19 restrictions in services.

And all this became very, very clear after we’d already sat on 18 months, two years of waiting list prior to COVID-19, and then those waiting lists, those specific waiting lists, doubled and trebled once we were coming out of the first lockdown. (Parent carer, A1_05)

Similarly, parent carers in this study reported receiving letters or phone calls to tell them that hospital appointments for speech and language therapy, occupational therapy, physiotherapy and clinical psychology reviews and assessments were cancelled, and they would be contacted again when an appointment could be offered. For some families, these appointments were still outstanding at time of interview. However, parent carers of children with paediatrician or specialist nurse managed conditions such as epilepsy continued access to services and support remotely throughout the pandemic and received communication confirming the continued access to services. For families who did attend in-person hospital appointments or who needed inpatient care during the pandemic, parent carers described how many hospital guidelines allowed only one parent to accompany a child. For parent carers of children with complex needs, this was seen as unfeasible and caused added stress while their child was in hospital. Several parent carers interviewed reported that exceptions were eventually made to allow two parent carers, due to the child’s needs. However, this often required significant negotiation with services.

…the hospital wanted just one parent to go in and [child]’s like very, very difficult at the time, screaming and he was throwing himself on the floor and it took a lot of convincing for them to let two parents in. (Parent carer, A4_003)

Parent carers raised the challenges experienced with equipment services, wheelchair and orthotics services during the pandemic. Most described long waits for equipment while services were closed, resulting in children not having the necessary equipment at home to move safely, and being in pain from wheelchairs or orthotics not fitting correctly.

And he grew and grew and grew, and he outgrew his equipment… But even when the service did open back up again, it was just so painfully slow, and they couldn’t get a hold of a wheelchair. They couldn’t get hold of this. It couldn’t get hold of that. (Parent carer, A4_14)

However, one parent carer noted that their equipment service appeared to be under less pressure as they were not operating as normal, and as a result they appreciated being given support to identify and order specialist equipment that they did not feel the service would have had time to provide under their normal workload. This parent carer asserted how important this experience was in helping their family to cope with a profound and extremely distressing decline in their child’s seizure control, and regression of developmental skills, resulting in lost speech and mobility.

But they [occupational therapist] really stood out in they knew that we were using toddler reins to hold (Daughter) up. And one of them had researched a specialist-made drop harness in America. They arranged for that to be ordered from America and actually also arranged for it to be customised so that we had a long handle so we could hold (Daughter) out…so that was incredible. So, I think we felt at the time that we probably had, whether we did have that bit more, that better service because their other patients weren’t in play, they weren’t having to juggle such a heavy caseload. (Parent carer, A1_03)

The widespread closure of services meant many families could only access services using telehealth. Parent carers reported that medical services had cancelled appointments, but most had moved swiftly across to telehealth delivery and were able to manage continued care with a familiar medical professional or specialist nurse. Advantages of telehealth included not having to take time out of work and school to attend in-person appointments, and not always needing to have the child present.

It was telephone appointments [with paediatrician]. Do you know what in some aspects they were better. Just the fact of not having to get my son into that building…and it also meant as well, not pulling them out of school, not having to change routine with them if they don’t need to see them face to face… It worked a lot better and some of the time they’ve actually given me the option since to carry on with video appointments or if they do need to see them to weigh either one of them, it’s a case. That’s fine. We’ll come in because there’s a need for them to be there. (Parent carer, A3_12)

For parent carers with younger children, telehealth appointments were easier to manage, as their child could be very unsettled in a face-to-face clinical settings, making it difficult to concentrate on what was being said. While many parents were familiar with online platforms, some did not have access to a computer or could not use technology to be able to access online appointments or learning. Others spoke of interruption to telehealth calls due to lost connections. For example, some were trying to join multi-agency meetings about their child on a phone or using technology they were not confident with, which then affected how well they could engage with and be heard in discussions about their child’s care.

I’m middle-aged plus. So again, my tech knowledge was obviously very, very limited as well as having old tech. I’d rather pick up a phone or something. I find it very difficult, but I do also feel that the services directly, were able to exploit my lack of technical knowledge to gain whatever they needed to… they promised to me help and I didn’t get it. I mean, one of the women said let’s see if we can get you an old computer. And we’ll just put such and such on it for you. Well, that was two years ago. I'm still waiting, you know. (Parent carer, A1_01)

While continued care was mostly acceptable via telehealth, new referrals and appointments were often regarded by parent carers as not working well using telehealth. Some parent carers reported professionals did not have details on their child when they met for telehealth appointments or were unable to understand the communication abilities and preferences of their child. Parent carers also reported challenges in engaging their child in either phone or video appointments due to difficulties with communication, attention or the child’s discomfort with these methods.

[Child] doesn’t really engage in the whole Zoom thing. And it’s not the same as like a face to face so… She[child] doesn’t do talking on zoom, but if you did an activity with a start, a finish, a middle and an end, [child] can stick with that because she knows her routines, whereas if it was just like a right [child], your teacher’s talking to you now, she’d be like well what are we doing? What’s happening? It wasn’t working. (Parent carer, A3_05)

Alongside telehealth appointments, parent carers also discussed the online resources they were signposted to by service providers (eg, physiotherapy and speech and language resources), while they waited for referrals to be accepted, or immediately after receiving a diagnosis. Parent carers felt when used without additional information from a professional, these resources fell short of providing adequate knowledge to enable intervention.

During the pandemic, parent carers described being a parent carer, teacher and therapist as they took on a day and night role caring for their child. Parent carers reported taking their child’s blood pressure and weight for medical services health reviews and to inform use of repeat prescriptions. Others reported trying to maintain their child’s physiotherapy programmes and speech and language activities at home, alongside home-schooling. They described multiple challenges achieving these tasks including being restricted by limited space at home, equipment, confidence and finding time among all the other family and work demands. A parent carer and young person spoke of their concern that while they had continued to do some physiotherapy they did not want to ‘*do more harm than good*’ (Young person, A4_09). Some parent carers described how hard it was to maintain everything over a long period of time, and how challenging it was engaging their child in activities with them.

They [physio] talked to me and said ‘Okay, what do you have at home in terms of equipment for [child] and let’s work with what you’ve got’. I said to them that we’ve got a mini trampoline, we’ve got a wobble board, we’ve got, stretching exercise bands and then they designed a program for the equipment that we’ve got.…. In terms of sustaining that over a long period that’s where the problem came in. For instance, now, you know, we’ve still got a need for [child], to have these exercises. He’s got very bored of me doing it with him. (Parent carer, A3_01)

Self-management/Parent carer management of care partly filled a gap but was not seen as suitable or sustainable long-term. Parent carers and children and young people reported difficulties in the transfer between services.

I think it messed it [transition to adulthood] up, to be honest, because where she was, she was set up for transitioning to adulthood or preparation. And it was all started, all the professionals were, when was the meeting? I think it was sort of around March, April, when the pandemic first started, or it got locked down. So, we had everyone, it was all arranged and then that got cancelled. And then, it just got delayed, and I feel it messed it up, to be honest. (Parent carer, A2_02)

Delays and disruption to the transition process was mostly referred to in relation to moving into new schools and from school into adult/postschool services. Parent carers and young people reported being unable to visit schools and colleges, which prevented them from seeing in-person the associated specialist support systems that would be needed. This made it difficult to identify and select appropriate provision.

Basically, I just sorted it myself, but it was obviously not ideal, not being able to look around school, I mean there was no choice really, so it was fine for us, but I guess if people had a choice of schools and they really wanted to see. I think we went around the school she was at after school hours, obviously not during lockdown but, when it opened. But it’s very hard to get an idea of what a school’s like when it’s completely empty and there’s no kids. (Parent carer, A3_03)

Moves from mainstream to specialist school settings were reported as positive as specialist providers had greater experience and understanding of supporting transition. Returning to school after lockdown was reported to be very difficult for some young people and there was little support for this return to the school routine. For some children and young people, this resulted in an escalation of distressed behaviours. Families who had children aged 18–19 years reported extreme difficulties in managing their child’s transition to adult services during the pandemic. Families experienced a sudden loss of children’s social care and preparation for transition, but adult social services were closed, leaving them with nowhere to go for support.

### Impact of service change

The loss of formal respite and short breaks provision was described by parent carers as having a devastating detrimental impact on their families. Families struggled to cope with high levels of medical care, and physical and behavioural support. The simultaneous loss of informal respite and routine associated with school was a particular focus of parent carers. They reported that losing this routine and input caused significant distress to their children and the family.

He was very distressed most of the time and not sleeping and all kinds of things. It was very, very, very difficult and we were told we’d get some social care over that summer, and I phoned repeatedly in tears and asked for some help. And we got absolutely nothing… And then in January, February time 2021, (Son) had a complete catatonic breakdown and lost the ability to talk and lost the ability to move and became doubly incontinent. Couldn't eat. I mean, it was really very dramatic, and we still didn't get any support and people couldn't believe that somebody would get so dramatically worse so quickly. (Parent carer, A3_03)

Interviewer: What was terrible about it [lockdown]? A4C_002: Not seeing my nana and granda for three months. Only seeing them outside. (Young person, A4C_002)

Parent carers reported negative outcomes for children and young people’s physical, behavioural and emotional/mental health. Parent carers of children and young people with physical disability reported deterioration in deformity, mobility and increased pain and discomfort and were concerned that these problems would become chronic due to lack of physiotherapy.

She can’t use any equipment, so she can’t use standers, walkers, anything like that, and she used to be able to, and she used to be able to pull herself to standing. She can’t do that she’s lost that skill… I got the impression that nobody really is prepared to comment on the aftercare. (Parent carer, A4_015)

Parent carers reported that their children experienced increases in challenging or distressed behaviours, aggression and anxiety, which they often ascribed to the loss of routine and specialist support in school during the pandemic.

Parent carers and children and young people described deterioration in children’s mental health, which they felt was exacerbated by their inability to access support through Children and Young People Services or Child and Adolescent Mental Health Services.

It had an impact on my physical health and my mental health too. I had it in my mind that I would need more operations. I was angry and upset about the operations ‘cause they’re easily preventable. (Young person, A4_09)

Parent carers reported difficulties in accessing mental health support for their children prior to COVID-19, which led to very low expectations of mental health services, with many now feeling they will have to manage things on their own. It is also important to note that some families found benefits in their children being at home during lockdown, including reduced demands at school that promoted better behavioural and emotional health, and in some cases, less dysregulated behaviour.

It [small school classes] was good for [Child] because the school environment was less busy. So although he’s in a special school, he still struggles with the school environment, but because there were so few pupils in school, he actually enjoyed it. And they weren’t really doing anything particularly academic, so there weren’t too many demands being put on him as well, so he, he thrived in that environment. So that was good. So that was a big positive. Umm and definitely something for schools to learn from. (Parent carer, A3_04)

Many parent carers described the negative impact of the pandemic on themselves. Parent carers reported high levels of stress, but no support being available to help them to manage their child’s care and health or behavioural difficulties. Most parent carers interviewed felt isolated and alone during the pandemic. Their usual systems of peer support groups and family were lost. The extra precautions families took to stay safe added to the isolation they were experiencing from their wider family and from social activities.

I mean one of the things that people didn’t understand was that we couldn’t just go out in the car. People would take their kids out for drives, or for a walk, which wasn’t going to happen for us. We were even more isolated I would say. (Parent carer, A1_12)

I think at one bit I just cried, because I thought, I just don’t know how I’m going to manage. How am I going to manage this. You know, all my world stopped, and you think, coping with somebody like [child] it mentally drains you, so that were really hard. (Parent carer, A3_07)

There was not time to attend online groups while they were juggling their family’s needs and often work demands as well. Many continued to experience isolation, perhaps more keenly as restrictions were eased because, unlike other families, their child could not return to school and/or not return to previous levels of socialising due to real or perceived vulnerability. Many specialist social activities had still not reopened or continued to restrict numbers attending at the time of interviews.

Parent carers thought the impact of COVID-19 would be felt for a long time to come and the impact had been profoundly greater on their disabled children than on their other children. Some parents raised how the experience of COVID-19 lockdown had influenced decisions about their child’s future and the plans they had about where their child would live once they were no longer in full-time education. The experience of caring for their child full-time, and witnessing how challenging some behaviours and needs had become, led some parents to believe that the family home might not be a suitable long-term placement, as they felt unable to cope with the support needs.

Any thoughts we might have had of him staying at home are just not viable. Between his increased needs and our need for our life back and recovery from the last few years. (Parent carer, A1_02).

### Learning for future emergencies and resetting services

Parent carers and young people asserted the need to provide a child and young person-led and family-centred approach to the resetting of services. They requested clearer information and coordination in health and care and asked that networks work together centred around the children and young people.

I’d like health and education to stop making unilateral decisions about the care of my son and do more consultation and engagement, but I think that’s probably going to take more time. (Parent carer, A1_08)

They stressed the importance of multidisciplinary team working to identify the needs of the children and young people and that appropriate care should be accessed in a timely manner to enable the early intervention and holistic support needed. They advocated for adequate resources to meet their children’s and young people’s medical, therapeutic and care needs, as well as support for the family to manage and mitigate the increased demands and pressure they experienced.

And also the other thing is that when things like this happen that are dramatic, families with children with neurodisability are not able to cope with the reduced, they need extra services than they had before, not less, and then the same amount restored it’s not feasible or practical to expect people to manage on that. And if we’d had, if we'd had the same level going through the pandemic, we may not have had the breakdown and needed the extra support, whereas because that’s now happened, we now need a massively higher level of support than we did before, which is really short sighted. (Parent carer, A3_03)

Having experienced profound difficulties in accessing services during the lockdowns and restriction periods, one young person described initiating a multidisciplinary team meeting to properly address issues with a siloed service approach to his physiotherapy and equipment needs. This was the first time he had access to a multidisciplinary team meeting and advocated for this as standard. He also worked with equipment services to improve their provision. The young person and his parent carer felt this coproduction approach should be central to service design and delivery.

Families expressed they needed more flexibility in service provision going forward. While telehealth was perceived as something that should continue, parent carers and young people thought they needed to be able to choose the type of appointment (phone/video/in-person) that met their needs and their family circumstance. They also wanted to maintain the flexibility that they had experienced in using personal budgets more creatively and in response to their child’s particular needs.

But then what they did do, which was good, was they allowed us to use our direct payments flexibly. So, I got a lot of educational materials with direct payments. So having all that flexibility of people coming into the house eventually and being able to buy what worked for us actually did work really well. (Parent carer, A1_11)

In a future emergency, families strongly advocated that communication systems be established expediently to provide clear guidance to and from schools, services and families about updates and any changes to processes. Guidance must be communicated clearly and effectively to families.

Point of contact. So you know who do you contact, if there is an emergency, or not an emergency, but you know with (Son) so, for example, with his feet, you know there were days when, I mean, so basically, the bones here kind of grow in so his feet kind of go in like that. You know, through the pandemic I’m like, I don’t know, who do I speak to do this, do I take him to A&E? (Parent carer, A4_07)

Families need to know who to contact and how to contact them. Parent carers and children and young people advocated for services to work together and to provide unambiguous roadmaps to care that were adhered to and information provided on these road maps. Parent carers asserted the need to review decisions on closure of schools during an emergency. They believed that many children were more vulnerable from missing school than from becoming unwell with COVID-19. School closures placed children at risk of socio-emotional and mental health issues. Likewise, parent carers advocated for the protected provision of short breaks through residential respite or personal budget support workers in a future emergency to support family well-being. Prioritisation of services to disabled children in a future emergency was seen as necessary to mitigate the impact on children, young people and their families.

In recovering from the COVID-19 pandemic, once lockdowns and restrictions were lifted, parent carers emphasised the need for adequate and targeted funding to support children and young people in addressing mobility issues, learning loss and mental health impacts of COVID-19. They also called for a full assessment of the long-term effects of the lockdowns, restrictions and changes to services on disabled children and young people.

There’s also a thing about distributing burden fairly, cause among the burden, you know a lot of things keep hitting us, as opposed to the wider population. So, for instance, because the pandemic has hit us particularly hard, you would, the appropriate societal response would be to invest in the recovery services, like that’s why the respite care two years down the line. It seems a particular betrayal because. By that point, you know the damage. You know the need. You know, the obligation to put things right. And yet you still haven’t done the job. (Parent carer, A1_02)

They also advocated for investment in promoting and supporting parent carer health and well-being to help them recover from and manage the ongoing pressures of caring for disabled child(ren) and their families.

## Discussion

This study offers detailed and profound insights into the experience of parent carers and young people with neurodisability during the lockdowns and restrictions of the COVID-19 pandemic, and the experiences of families following the lifting of all restrictions in the UK after February 2022. Most families experienced extensive and long-term disruption to services they previously accessed routinely and depended on. Parent carers found it difficult to find out what was happening to services in their area, were unsure about how to keep their child and family safe from infection and faced long periods of uncertainty around when service access would resume post lockdown. The introduction of telehealth had mixed success. For some, telehealth was an effective interim measure for some medical care and continued contact with education, social care services and therapy services. However, many reflected that rapid changes to services had not considered how children and young people with neurodisability could engage with care remotely.

Parent carers’ descriptions of feeling abandoned by therapeutic, equipment and assessment services, school-based services and social care and support are similar to those reported previously.^11^,^13^ Participants’ experiences contrast with the concerted efforts made by professionals in our parallel study to contact families and establish effective methods of sharing often rapidly changing information.^17^ Clearly, lines of communication did not work for all families and efforts to reset services and plan for future emergencies should involve agreement between providers and families on how information will be shared.

Similar to other recent research,^25^,^29^ parent carers and professionals in our research^17^ agreed that some medical services moved swiftly and effectively to telehealth provision but that on the whole telehealth for therapy services was less successful. Although telehealth delivered in the pandemic allowed some interaction between families and services to continue, the long-term use of telehealth requires further innovation to increase the range of services to meet the needs of a wider range of children and young people. However, it is important to note that, even with innovations, there are still services (eg, orthotics, assistive technology) and circumstances (eg, safeguarding, digital poverty) where the use of telehealth may be insufficient or inappropriate.

Like other research, our findings indicate the major disruption to families from the lockdowns and restrictions put in place due to the COVID-19 pandemic.^8 13^ The long-term impacts are yet to manifest, and many families of children and young people with neurodisability continue to experience, physically and mentally, negative impacts of lockdowns and withdrawal of or limited access to services. Resetting services is taking place at a time of greater need arising from problems exacerbated by the pandemic and growing waiting lists that have built up in the UK for many reasons, including loss of assessment opportunities and potentially inappropriate referrals.^12 30^ This situation is reflected across the National Health Service (NHS) and its links with social care whereby prepandemic weakness exposed vulnerable populations to further inequalities and avoidable harms.^31^ Crucially, unprecedented levels of vacancy exist across the children’s services workforce.^32 33^ The SEND and AP Improvement Plan^34^ may offer an opportunity to test how service provision can be redesigned to meet local needs equitably; however, this requires concerted collaboration and co-production between commissioners from all agencies, services and families.

Planning for future emergencies should assess the short-term and long-term impacts of service change on children and young people and their families.^35^ The health and well-being of parent carers is vital for children’s long-term recovery from the impacts of the COVID-19 pandemic. There was a pressing need to support the health of parent carers before the pandemic.^36 37^ The profound and prolonged additional pressure from service disruption compounded the problems. Addressing health of carers is already a priority for the NHS^38^; the health needs of parent carers must not be neglected in the recovery from the COVID-19 pandemic or forgotten in any future emergencies. Upscaling specific parent carer-focused health promotion programmes, such as healthy parent carers, offer considerable potential for addressing the recognised risks of physical and mental health problems.^39^ Health professionals may lack confidence and skills to support the needs of parental mental health and well-being within their professional roles, and there are evaluations of tailored training for key workers and managers with encouraging results.^40^

The findings from this study present an overview of a range of parent carer experiences across different and diverse areas of England during and following the restrictions and lockdowns associated with the COVID-19 pandemic. In the analysis for this paper, we did not seek to compare experiences across areas. However, through the process of arranging remote interviews and through the interviews themselves, it was clear that digital poverty had an impact on how much families could find the information they needed about service access, and on how they could engage with services. Families had a range of financial resources to be able to access equipment (eg, trampolines, craft supplies) to support their child, access private assessments to reduce wait times and access to open spaces for safe social distancing. In line with other research that has highlighted the inequalities and disproportionate impact of the pandemic on disabled people,^41^,^43^ many of the parent carers highlighted how even when the rest of the country was ‘back to normal’, they were still experiencing continued disruption and feelings of being left behind due to services for their children not being prioritised during the lockdowns or the recovery.

The timing of the interviews (November 2021 to July 2022) allowed for reflection on how services had changed over several stages of the pandemic as we came out of the lockdown period, and allowed for reflection on what was and was not effective in supporting families. Although, this also means that the interviews were collecting a post hoc account of families’ experiences and feelings as the pandemic unfolded which may be reflected on differently at this time point, compared with at the actual time of events. However, a limitation to our work is that, despite various attempts to include young people’s experiences in this research as well, we were not able to recruit as many young people as we hoped to participate in interviews or focus groups. We believe it was important to include the voices of young people we were able to interview, despite the small number. Reflections from parent carers and young people involved in our PPI work suggest the low interest from young people might be because experiences during the pandemic were stressful and reflecting on these in an interview would be upsetting. Some young people may have just wanted to move on from experiences of the pandemic, and for some taking part in an interview may not have been desirable. Purposive sampling was used to gather broader views from parent carers of children with differing diagnoses; however, registrations of interest were largely from parent carers of autistic children, which may have slightly biased the experiences gathered compared with those of parent cares of children with other diagnoses. However, interviews still covered parent carers of children with a wide range of needs and comorbidities. Also, most interviews were with mothers, potentially reflecting that mothers are often the primary caregiver of disabled children^44^ and have been found to be disproportionately affected by the COVID-19 pandemic.^45^ We did not collect the ethnicity of the families interviewed, however most of the families interviewed were White British. As part of recruitment process, several organisations were approached to support the inclusion of families from diverse ethnic backgrounds and to ensure the voices of underserved groups were represented.^46^ However, despite our best efforts to focus on this, we were unable to recruit from these groups.

The findings from these interviews have been used alongside interviews with health, education and social care professionals^17^ to make recommendations on service delivery in future emergencies and to reflect on lessons learnt.^47^ These recommendations were presented in a Delphi survey to key stakeholders (parent carers, young people, commissioners, managers and frontline professionals) to reach a consensus on a set of recommendations.^48^ These recommendations outline clear practice and policy recommendations for delivering effective services to disabled children in future emergencies.

## Conclusion

COVID-19 and decisions made about education, health and social care policy and services led to significant changes in provision for children and young people with neurodisability. These had deleterious, sometimes devastating, impacts on young people’s health, function and participation, and continues to do so. Multidisciplinary team working and flexible service delivery, centred around the needs of children and young people, were seen as essential for future provision and emergencies. Services need to work together around a child or young person to be able to meet their therapy, equipment and social support needs holistically and this needs to be achieved through effective collaboration of services and integrated practice. In any future emergency, there should be tested and resilient systems in place to prioritise safe access to in-person care and support where necessary, as well as effective remote provision to mitigate the impact of service disruptions on both children and young people, and on parent carer health and well-being.

## supplementary material

10.1136/bmjopen-2024-085144 online supplemental file 1

10.1136/bmjopen-2024-085144 online supplemental file 2

## Data Availability

Data are available on reasonable request.
