# Supplementary material for: Parent carer and disabled young people’s perspectives on the impacts of changes to service provision for children and young people in England during the COVID-19 pandemic: a qualitative study
Source: BMJ Open. 2024 Nov 27;14(11):e085144. doi: 10.1136/bmjopen-2024-085144 (PMC11603685; doi:10.1136/bmjopen-2024-085144)
Supplement: online supplemental file 1 [file bmjopen-14-11-s001.pdf]

Resetting Services to Disabled Children

Interview Topic Guide: Parents

Introduction

Aim of the project is to learn from the changes made to services in response to COVID-19 and find out how services for disabled children could be remodelled to be effective and acceptable. We want to hear from families about the changes in services they experienced, what worked and what didn't. There aren't any right or wrong answers and if there are any questions you do not feel comfortable answering that is okay, you will not have to. Anything you tell us will be confidential. If we use quotes from interviews in reports, they won't be identifiable to you.

Consent to record the interview

You've previously been sent information about the project and filled in the consent form; thank you for doing that. Are you still happy for us to audio-record the interview?

[Explanation if needed: Recording interviews means that we have a more accurate representation of the interview for our analysis and we can focus our attention on your during the interview, rather than taking lots of notes].

Interview questions

Thinking back to December 2019, could you briefly tell me which services your child was receiving before the pandemic?

How many services? Prompts:

Medical services;

Therapy and psychology;

Social care (social workers, short breaks);

Education

Early years if appropriate

How often did your child attend/received these services?

For how long have they been involved with these services?

What changes in services did you and your child experience over the covid-19 pandemic from the first lockdown in March 2020 to now?

*Thinking back over what has happened since the first lockdown in March 2020 and how services have changed, which changes do you think we should **keep**? What we should carry on doing?*

Why is that? Why has it worked well?

What do you think we should ***stop*** *doing? Why is that, why has it not worked?*

What do you think we should ***start*** *doing? How would this help? Have you heard about this intervention / process being used in other places?*

Prompts:
Changes to each service for each question above – positive and negative impacts on physical and mental health of child, family; parental involvement in therapy programmes programmes; social participation; quality of life; impacts on siblings and wider family; technology and other resources required; learning and behaviour change required; assessment and diagnosis vs management and ongoing programmes

Services to include:
Medical services – hospital and community
Therapy and psychology
Social care (social workers, short breaks)
Education – did child stop attending school – when and why
Early years if appropriate
Coordination of services
Communication with services

Close

Thank family for participation

Next steps: interviewing other families and providers of services, bring information together what has and hasn't worked for which groups and why. Future survey to agree recommendations. Will contact family about that using details they previously supplied. Family can get in touch any time about the study. Updates will be on website.
