# Supplementary material for: Parent carer and disabled young people’s perspectives on the impacts of changes to service provision for children and young people in England during the COVID-19 pandemic: a qualitative study
Source: BMJ Open. 2024 Nov 27;14(11):e085144. doi: 10.1136/bmjopen-2024-085144 (PMC11603685; doi:10.1136/bmjopen-2024-085144)
Supplement: online supplemental file 2 [file bmjopen-14-11-s002.pdf]

Resetting Services to Disabled Children

Interview Topic Guide: Young Person

Introduction

Services like speech and language therapy and physiotherapy changed because of COVID-19. We are asking young people and families about the changes. There aren't any right or wrong answers. If there are questions you don't want to answer, you don't have to. You can stop the meeting at any time. Anything you tell us will be confidential. People will not know that you took part in the research.

Consent to record the interview

You've already had information about the project and filled in the consent form; thank you for doing that. Are you still happy for us to audio-record the interview?

[Explanation if needed: Recording interviews means that we have a more accurate representation of the interview for our analysis and we can focus our attention on your during the interview, rather than taking lots of notes].

Interview questions

Can you tell me who helps you? Which therapists and services?

Prompts:

Medical services;

Therapy and psychology;

Sensory – VI, HI

Social care (social workers, short breaks);

Education

How did therapy and services you get change during COVID?

Prompt: where sessions take place; how professionals communicate with child (e.g. phone, video); how many professionals are seen (e.g. all services via one key professional)

Did you go to school during COVID? What happened to services there?

What was good about the changes? Why did that help?

What didn't work well? Why was it not good for you?

Are there other things that could help make therapy and other services easier for you and your family?

Prompts:
Setting - Online; phone sessions; face to face
Model of care – number of professionals seen (interdisciplinary working; professionals combining sessions eg physio and OT doing a session with a child together; same goal across services) timing of sessions, frequency of sessions
Parent involvement in therapy programmes programmes

Close

Thank young person and family for taking part

Next steps: interviewing other families and providers of services, bring information together what has and hasn't worked for which groups and why. Future survey to agree recommendations. Will contact family about that using details they previously supplied. Family can get in touch any time about the study. Updates will be on website.
