# Supplementary material for: Parent carer and disabled young people’s perspectives on the impacts of changes to service provision for children and young people in England during the COVID-19 pandemic: a qualitative study
Source: BMJ Open. 2024 Nov 27;14(11):e085144. doi: 10.1136/bmjopen-2024-085144 (PMC11603685; doi:10.1136/bmjopen-2024-085144)
Supplement: Supplementary file 3 [file bmjopen-2024-085144.reviewer_comments.pdf]

PEER REVIEW HISTORY

BMJ Open publishes all reviews undertaken for accepted manuscripts. Reviewers are asked to complete a checklist review form and are provided with free text boxes to elaborate on their assessment. These free text comments are reproduced below.

ARTICLE DETAILS

Title (Provisional)

Parent carer and disabled young people's perspectives on the impacts of changes to service provision for children and young people in England during the COVID-19 pandemic: a qualitative study.

Authors

Merrick, Hannah; Driver, Helen; Potts, Lily; Exley, Catherine; Allard, Amanda; Morris, Christopher; Parr, Jeremy R; Pennington, Lindsay; Resetting Services Group, .

VERSION 1 - REVIEW

**Reviewer**	**1**
**Name**	**Langford, Rebecca**
**Affiliation**	**University of Bristol, Bristol Medical School**
**Date**	**22-Mar-2024**
**COI**	**No competing interests**

Thank you for asking me to review this very interesting paper on the changes to service provision for young people with neurodisabilities during COVID. This is a well written and thoughtful paper, highlighting the often devastating impact of the pandemic response on some of the most vulnerable children in society. The study was well-conducted with excellent input from public contributors throughout the study. The analytical approach is clearly described and the themes well-articulated.

I would recommend this for publication in your journal but note a few suggestions below which I believe might improve the paper.

Quotes

There are some really poignant quotes included in the paper. But I must admit to finding it very hard to read the paper when I had to keep flicking back and forth from the main text to the boxes. Presumably this has been done due to the journal's word count but if there was a way to embed the quotes in the text, it would make for an easier and more powerful read.

I also think most of the quotes are overly long and could easily be edited down to aid readability while still retaining essential content and meaning. (I know there is an argument

for keeping naturalistic language in quotes but since this study is primarily focusing on content rather than linguistic analysis, I don't think this applie here). Quote 2a for example could easily be edited down as follow:

"[Child A] obviously wasn't having no occupational therapy or speech therapy because he wasn't in school… He just wasn't having no therapy and he wasn't having no speech [therapy]... I think that definitely affected … the way he was moving and obviously affected his speech because he was at home

with Mum and Dad."

Theme 4/Discussion

I suspect much of what the parent carers advocate for in Theme 4 is no different to what they would have said pre-pandemic. Issues around poor communication and lack of coordination in SEND / disability services is nothing new – though clearly exacerbated during Covid. For me, the most important (and novel) part of theme 4, was therefore their advocating for more and better resources to mitigate the significant detrimental impacts on these vulnerable children (and their carers), yet this issue felt somewhat buried. Similarly, I think the discussion would benefit from being stronger and clearer in drawing out the implications of these findings for government policy for disabled children. There are many sensible suggestions arising from this research – a table articulating they key learning points and policy recommendations would be beneficial.

Finally, I also think you could add in to paragraph 2 of p19 some acknowledgement of how the burden of care during the pandemic overwhelmingly fell on women's shoulders and that this needs to be acknowledged in terms of specific support for women.

Minor points

• The abstract does not mention that children and young people were included as interviewees.

• Table 1 is a lovely description of the PPIE input. However it mentions a 'mapping review' which is not referred to anywhere else. To avoid confusion I would suggest explaining what this was (and how it related to the current project) or removing this.

• P6 I think the term is 'Education Other Than At School' (not 'otherwise')

• Table 2 – apologies if I have missed this, but as several families had more than one neurodisabled child, did you collect gender, age, category of need data on each child in the family, or an index child. If the latter, how was the index child chosen?

• The enormous impact on the wellbeing of parent carers during this time is really powerful and rightly discussed in the discussion section. Did you collect any data on parent carers about how many were in paid employment (part or full time) during this period or if any were furloughed?

• The term 'short breaks' might need explaining (e.g. I assume this is short respite care?)

• Some acronyms have not been defined e.g. SALT. It would aid the reader if the authors could provide a list of acronyms used within the paper at the end.

• There are some inaccuracies in the transcribed quotes e.g. 1g “what the class is non essential” – presumably this should be “what is classed as non essential”? And later, “covid words” instead of “wards.” Linked to my comment above, I would suggest checking all quotes for clarity and accuracy throughout.

• You use both the terms “autistic children” and “children with autism” in the paper. Though people vary in their preferences, the autistic community generally prefers “autistic” rather than “with autism”.

• There is some repetition and overlap between theme 1 and 2 (e.g. fourth paragraph of p11 repeats issues around communication of appointments).

• I would suggest swapping round the two quotes provided in 2b so they better match up with the text on p11.

• Some quotes are lacking a participant identifier of parent carer or young person.

• You note the timings of the interviews (in Nov 21- July 22) as a benefit but I think it would only be fair to acknowledge this does also pose a limitation to your research too. What you have here is a post-hoc assessment of the pandemic, rather than their actual thoughts and feelings as the pandemic unfolded. Participant recollection and interpretation of events will have changed over the ensuing time period.

**Reviewer**	**2**
**Name**	**Milic, Jelena**
**Affiliation**	**Erasmus MC**
**Date**	**02-May-2024**
**COI**	**non**

:Dear authors,

Congratulation on the choice of topic. This is an interesting research question and it is good that opened this research problematics. Overall the idea of the study is good but there are several major point that can benefit from revision. Please find enclosed my general comments and I hope you will find them useful to revise the current manuscript.

Please update the keywords s existing look inappropriate:

COVID-19, Developmental neurology & neurodisability < PAEDIATRICS, Health Services

1. ABSTRACT - You need to elaborate from the start what is tee exact change that makes this substantial difference,, t is highly unprofessional to have such a strong opening statement,

that is actually a reason to do the study without arguments . you need to back up this strong statement with precise statement of changes.

2. Sampling:, Though you mention in single line in weaknesses how it was difficult to get more participants , you need to address more in the methods and discussion that the sample size is appropriate and representative of the target population. Consider including a diverse range of participants in terms of age, gender, ethnicity, and socioeconomic background to capture a comprehensive understanding of the experiences in additional sensitivity analyses and try to do comparing with the original findings.

3. Recruitment: Describe the recruitment process in more details detail to ensure transparency and to assess the potential for bias. Consider including detailed inclusion graph

4. Data collection: Use more details to describer the phone interviews, focus groups, and observations, to gather rich and comprehensive data gathering. This is always tricky as you are getting self perceived answered so try to explain how you designed the interviews in order to get the more objective answers.

5. Ethical considerations: mention more about obtained informed consent from participants and ensuring in the final draft their confidentiality and anonymity. As this is a highly sensitive set of diagnosis and minors are mostly in question via parents, this emphasize is considered important.

6. Data analysis: Try to use a rigorous and systematic approach to analyze the qualitative data. Consider using established qualitative analysis methods, such as thematic analysis or grounded theory, to identify common themes and patterns in the data. This might work better than current analysis, so try re-analyze .

7. Reflexivity: Acknowledge the potential influence of the researcher's background, biases, and assumptions on the research process and findings. Reflect on your own positionality and how it may impact the interpretation of the data.

8. Trustworthiness: Enhance the trustworthiness of the study by using techniques such as member checking, peer debriefing, and triangulation of data sources to ensure the credibility, transferability, dependability, and confirmability of the findings.

9. Reporting: More clearly describe the study design, methods, and findings in the research report. Provide sufficient detail to allow for replication and ensure transparency in the research process.

10. Add the sections about clinical implication of findings and future research directions.

VERSION 1 - AUTHOR RESPONSE

Reviewer: 1
Dr. Rebecca Langford, University of Bristol

Comments to the Author:

Thank you for asking me to review this very interesting paper on the changes to service provision for young people with neurodisabilities during COVID. This is a well written and thoughtful paper, highlighting the often devastating impact of the pandemic response on some of the most vulnerable children in society. The study was well-conducted with excellent input from public contributors throughout the study. The analytical approach is clearly described and the themes well-articulated.

I would recommend this for publication in your journal but note a few suggestions below which I believe might improve the paper.

Thank you.

Quotes

There are some really poignant quotes included in the paper. But I must admit to finding it very hard to read the paper when I had to keep flicking back and forth from the main text to the boxes. Presumably this has been done due to the journal's word count but if there was a way to embed the quotes in the text, it would make for an easier and more powerful read.

Thank you. Yes, in order to stay as close as possible to the journal word count, we made the decision to put the quotations in boxes so that all the findings can be presented in the necessary detail to give the full narrative of the parent carers' and young people's experiences.

I also think most of the quotes are overly long and could easily be edited down to aid readability while still retaining essential content and meaning. (I know there is an argument for keeping naturalistic language in quotes but since this study is primarily focusing on content rather than linguistic analysis, I don't think this applies here). Quote 2a for example could easily be edited down as follow:

"[Child A] obviously wasn't having no occupational therapy or speech therapy because he wasn't in school… He just wasn't having no therapy and he wasn't having no speech [therapy]... I think that definitely affected … the way he was moving and obviously affected his speech because he was at home with Mum and Dad."

Thank you. We have gone through the quotations and edited them down to present the key information, which hopefully aids readability.

Theme 4/Discussion

I suspect much of what the parent carers advocate for in Theme 4 is no different to what they would have said pre-pandemic. Issues around poor communication and lack of coordination in SEND / disability services is nothing new – though clearly exacerbated during Covid. For me, the most important (and novel) part of theme 4, was therefore their advocating for more and better resources to mitigate the significant detrimental impacts on these vulnerable children (and their carers), yet this issue felt somewhat buried.

We agree that some of these issues were present pre-pandemic, nonetheless the exacerbation during the pandemic provides an opportunity to reinforce the disparity between need and resources. We have changed some text in theme 4 on page 18 emphasis this point.

"They stressed the importance of multidisciplinary team working to identify the needs of the children and young people and that appropriate care should be accessed in a timely manner to enable the early intervention and holistic support needed. They advocated for adequate resources to meet their

children and young people's medical, therapeutic and care needs, and also support the family to manage and mitigate for the increased demands and pressure that they experienced (Box 4b)."

Similarly, I think the discussion would benefit from being stronger and clearer in drawing out the implications of these findings for government policy for disabled children. There are many sensible suggestions arising from this research – a table articulating they key learning points and policy recommendations would be beneficial.

Thank you. We have added further detail to the discussion on page 21, on how the findings from this paper and the wider research programme have been used to inform policy and make recommendations for the delivery of services in a future emergency.

"The findings from these interviews have been used alongside interviews with health, education and social care professionals (17) to make recommendations on service delivery in future emergencies and to reflect on lessons learned (46). These recommendations have been presented in a Delphi Survey (47) to key stakeholders (parent carers, young people, commissioners, managers and front-line professionals) to agree a set of recommendations. These recommendations outline clear practical and policy recommendations for delivering effective services to disabled children in future emergencies."

Finally, I also think you could add into paragraph 2 of p19 some acknowledgement of how the burden of care during the pandemic overwhelmingly fell on women's shoulders and that this needs to be acknowledged in terms of specific support for women.
Thank you. We have added acknowledgement of this to the discussion on page 21.

"Also, most interviews were with mothers, potentially reflecting that mothers are often the primary caregiver of disabled children (43) and have been found to be disproportionately affected by the COVID-19 pandemic (44)."

Minor points
• The abstract does not mention that children and young people were included as interviewees.

Thank you for highlighting this. Details of the children and young people interviewed have been added to the abstract.

"Nine young people (aged 8-16 years; 8 males, 1 female) were interviewed; two individually, three in a focus group, and four with their parent carer."

• Table 1 is a lovely description of the PPIE input. However it mentions a 'mapping review' which is not referred to anywhere else. To avoid confusion I would suggest explaining what this was (and how it related to the current project) or removing this.

Thank you. We have updated the description in Table 1 to focus on the PPIE work for this part of the research study.

"The advisory groups were initially asked to provide feedback on the interview tools. Later, findings from the interviews and interpretations were presented to the groups and discussed. Meeting materials were adapted to enable access for all group members including those with communication support needs. In the final meetings for each group, the interview findings were shared and discussed."

• P6 I think the term is 'Education Other Than At School' (not 'otherwise')

Thank you, this has been changed to ‘other’

• Table 2 – apologies if I have missed this, but as several families had more than one neurodisabled child, did you collect gender, age, category of need data on each child in the family, or an index child. If the latter, how was the index child chosen?

Gender, age and category of need were collected for each child in the family. The figures in table 2 cover the details of all the disabled children in the families interviewed. We did not report only one index child.

• The enormous impact on the wellbeing of parent carers during this time is really powerful and rightly discussed in the discussion section. Did you collect any data on parent carers about how many were in paid employment (part or full time) during this period or if any were furloughed?

We did not systematically collect this data from the parent carers interviewed and therefore are not able to report on these aspects.

• The term ‘short breaks’ might need explaining (e.g. I assume this is short respite care?)

Thank you. We have added further explanation on page 9 where the term is first used.

“Parent carers reported closure or stopping of multiple services, including short breaks (i.e. residential centres, day care services, overnight breaks), school….”

• Some acronyms have not been defined e.g. SALT. It would aid the reader if the authors could provide a list of acronyms used within the paper at the end.

Thank you. We have gone through the document and made sure acronyms are defined at first use.

Page 6: ADHD – Attention deficit hyperactivity disorder
Page 10, explained SALT [speech and language therapist]

• There are some inaccuracies in the transcribed quotes e.g. 1g “what the class is non essential” – presumably this should be “what is classed as non essential”? And later, “covid words” instead of “wards.” Linked to my comment above, I would suggest checking all quotes for clarity and accuracy throughout.

Thank you. All quotes have now been checked for clarity and accuracy.

• You use both the terms “autistic children” and “children with autism” in the paper. Though people vary in their preferences, the autistic community generally prefers “autistic” rather than “with autism”.

Thank you for highlighting this. This has been changed to autistic children on page 11 to be consistent throughout the paper.

• There is some repetition and overlap between theme 1 and 2 (e.g. fourth paragraph of p11 repeats issues around communication of appointments). I would suggest swapping round the two quotes provided in 2b so they better match up with the text on p11.

Thank you. This change has been made.

• Some quotes are lacking a participant identifier of parent carer or young person.

Thank you for pointing this out. All quotes now have a unique participant identifier.

• You note the timings of the interviews (in Nov 21- July 22) as a benefit but I think it would only be fair to acknowledge this does also pose a limitation to your research too. What you have here is a post-hoc assessment of the pandemic, rather than their actual thoughts and feelings as the pandemic unfolded. Participant recollection and interpretation of events will have changed over the ensuing time period.

Thank you. We have added a sentence to the discussion (page 19) acknowledging that the timing of interviews was a potential limitation to the study.

"Although, this also means that the interviews collected post-hoc accounts of families' experiences and feelings as the pandemic unfolded which may be reflected on differently at this time point, compared to at the actual time of events."

Reviewer: 2
Dr. Jelena Milic, Erasmus MC, Institut za javno zdravlje Srbije Dr Milan Jovanovic Batut
Comments to the Author:
Dear authors,
Congratulation on the choice of topic. This is an interesting research question, and it is good that opened this research problematics. Overall, the idea of the study is good but there are several major points that can benefit from revision. Please find enclosed my general comments and I hope you will find them useful to revise the current manuscript.

Please update the keywords s existing look inappropriate:
COVID-19, Developmental neurology & neurodisability < PAEDIATRICS, Health Services

1. ABSTRACT - You need to elaborate from the start what is the exact change that makes this substantial difference, it is highly unprofessional to have such a strong opening statement, that is actually a reason to do the study without arguments. You need to back up this strong statement with precise statement of changes.

Thank you. We have edited the first sentence of the abstract to provide more detail on the services changes experienced by families to support the aims of the paper. The objective now reads as:

"In England, the delivery of health, education and social care services changed substantially. Some services closed, some had reduced capacity and there was a shift to the use of telehealth. The aim of this study was to understand how families of children and young people with neurodisability experienced these service changes, what did or did not work well for them and what impact the service changes had on them."

2. Sampling: Though you mention in single line in weaknesses how it was difficult to get more participants, you need to address more in the methods and discussion that the sample size is appropriate and representative of the target population. Consider including a diverse range of participants in terms of age, gender, ethnicity, and socioeconomic background to capture a comprehensive understanding of the experiences in additional sensitivity analyses and try to do comparing with the original findings.

We have reordered the participant and recruitment section in the methods on pages 3-4 to make the sampling methods clearer. With regards to the sample size, we describe a target number of participants based on previous research. The concept of defined or exact sample size for qualitative research is debateable and the purpose is to seek information power which can vary depending on the nature of the question under investigation (see Malterud, Siersma, & Guassora, 2016). As is common practice in qualitative research, the adequacy of the final sample size was continuously evaluated during the research process to ensure sufficient data were collected to robustly answer the research questions and gather a broad range of experiences. (Barbour, 2013, pg 66-69; Green & Thorogood, 2018) pg 102-104).

Purposive sampling was used to gain "information-rich cases for in depth study" rather than aiming for generalisability or full representativeness (Patton, 2002, pg. 230). Ideally, we would have preferred to recruit a more diverse sample based on age of the child, type of disability and family circumstances, but this was not possible given the time constraints of the project and time of investigation. In Table 2 we have presented transparently that overall, we were able to recruit families with a diverse child age range and category of need. We have added to the limitations about the potentially underserved groups whose voices we were unable to capture (page 21).

"We did not collect the ethnicity of the families interviewed, however most of the families interviewed were White British. As part of recruitment, several organisations were approached to support the recruitment of families from diverse ethnic backgrounds and to try to include the voices of underserved groups (45), but despite best efforts to focus on this we were unable to recruit from these groups."

3. Recruitment: Describe the recruitment process in more details detail to ensure transparency and to assess the potential for bias. Consider including detailed inclusion graph

Thank you. We have added further detail in the participant and recruitment section about the recruitment process on pages 3-4. This includes further detail about how information about the study was cascaded to participants, how they registered an interest and how we selected participants to take part in the interviews from those who registered on the study website.

"The registration forms asked participants to provide the age of their child, their child's category of need, which region they lived in, which parts of the study they were interested in and their contact details. These details were used to identify eligible participants in each of the five areas that were interested in taking part in an interview. Due to the number of registrations with interest to take part in an interview, all those that selected an interest within the five areas were contacted to take part."

4. Data collection: Use more details to describe the phone interviews, focus groups, and observations, to gather rich and comprehensive data gathering. This is always tricky as you are getting self-perceived answered so try to explain how you designed the interviews in order to get the more objective answers.

All interviews with parent carers were a 1:1 interview between the researcher and the participant to gather their narrative of their experiences of service change and its impact during the COVID-19 pandemic. Sorry we omitted details of the young people's interviews; this has now been added. We did not carry out any observations. We have added further detail regarding the interview procedures on pages 4-5 and we have included a supplement that shows the topic guide used in the interviews to gather data.

“The interview topic guide (see supplement 1) included initial questions asking participants to describe the services they accessed prior to the pandemic, and how often they accessed them. This was used to then understand how each of these services changed during the pandemic with open-ended questions focusing on how and in what ways services changed for them during the pandemic, the impact of these changes on families, what worked well and did not work well, and what would have made substantive difference to improve the experience of children, parent carers and families during the pandemic. Most the young people were interviewed via video call with their parent carer. One small focus group was held with a group of young people in person at their college (for interview topic guide, see supplement 2). All interviews were transcribed verbatim during the interview using Microsoft Stream for Teams or Zoom. Transcripts were checked for accuracy, corrected, and anonymised, removing any identifying information about the family and child, and specifically named services and places.”

5. Ethical considerations: mention more about obtained informed consent from participants and ensuring in the final draft their confidentiality and anonymity. As this is a highly sensitive set of diagnosis and minors are mostly in question via parents, this emphasize is considered important.

Additional information added to page 4: “At the start of the interview, participants reconfirmed verbally consent to the recording of the interview, participation in the study, and understanding that transcripts will be anonymised for analysis to keep participants’ identities and others mentioned in interviews confidential.” And on page 5 “Transcripts were checked for accuracy, corrected, and anonymised, removing any identifying information about the family and child, and specifically named services and places.”

We have been very careful to only include quotations that are anonymised and do not identify a family or child in any way.

6. Data analysis: Try to use a rigorous and systematic approach to analyse the qualitative data. Consider using established qualitative analysis methods, such as thematic analysis or grounded theory, to identify common themes and patterns in the data. This might work better than current analysis, so try re-analyse.

We have added some additional detail to the analysis section on page 6 to describe our use constant comparison methods and have updated the references to support this approach to analysis. Constant comparison methods are a core component of grounded theory to draw out the emerging patterns and themes in the data. Framework analysis was then used to assist in organising and presenting this data.

“Data collection and analysis was iterative, following principles of the constant comparison method, a core component of grounded theory (19, 20). This iterative approach allowed us to capture quickly emergent themes directly from the data, systematically look at who is saying what in which contexts, and explore those themes and comparisons with the research team and our PPI advisors (21). Three researchers (HM, HD, LPo) analysed the transcripts. Analysis was assisted by the Framework approach (22). An analytical framework was developed through open coding of initial transcripts and with reference to the EPOC taxonomy (23) for service change codes. Data were charted using the framework matrix and then summarised by category.”

7. Reflexivity: Acknowledge the potential influence of the researcher's background, biases, and assumptions on the research process and findings. Reflect on your own positionality and how it may impact the interpretation of the data.

We have added further detail to the reflexivity section on page 5 to reflect on our own positionality and methods we have used to try to mitigate potential biases in the interpretation of the data.

"It is recognised that both HD and HM who led on recruitment, interviews and analysis are both white, female and university educated which may have impacted on who responded to the research invitation and our perspectives and interactions. To mitigate the potential biases introduced by our positionality, PPI engagement with stakeholders (parent carers and children and young people) provided feedback and critical appraisal throughout the research process, including findings from interviews. We have also strived to maintain transparency in reporting our methods and findings. The varied experience and diverse positioning of the research team and PPI engagement established trustworthiness in the research process (24)."

8. Trustworthiness: Enhance the trustworthiness of the study by using techniques such as member checking, peer debriefing, and triangulation of data sources to ensure the credibility, transferability, dependability, and confirmability of the findings.

We have provided further detail in the reflexivity section on page 5 on how PPIE was used to enhance the trustworthiness of the findings. "PPI engagement with stakeholders (parent carers and children and young people), from wider areas than recruitment and diverse backgrounds, provided feedback and critical appraisal throughout the research process, including findings from interviews. The varied experience and diverse positioning of the research team and PPI engagement established trustworthiness in the research process (23)."

We have also added further details about this to Table 1 in section 3.

9. Reporting: More clearly describe the study design, methods, and findings in the research report. Provide sufficient detail to allow for replication and ensure transparency in the research process.

We have added further details throughout the paper where possible within the word count limit of the journal to clearly describe the study design, methods and findings. The Standards for Reporting Qualitative Research (SRQR) submitted now includes page numbers and paragraph numbers to identify where the necessary information is reported in the manuscript.

10. Add the sections about clinical implication of findings and future research directions.

Thank you. We have added an additional paragraph to the discussion (page 21) that describes the work that followed this and how the findings have been used to inform practice and policy recommendations for the delivery of services to disabled children in future emergencies.

"The findings from these interviews have been used alongside interviews with health, education and social care professionals (17) to make recommendations on service delivery in future emergencies and to reflect on lessons learned (19). These recommendations have been presented in a Delphi Survey (47) to key stakeholders (parent carers, young people, commissioners, managers and front-line professionals) to agree a set of recommendations. These recommendations outline clear practice and policy recommendations for delivering effective services to disabled children in future emergencies."

VERSION 2 - REVIEW

**Reviewer**	**1**
**Name**	**Langford, Rebecca**
**Affiliation**	**University of Bristol, Bristol Medical School**
**Date**	**16-Jul-2024**
**COI**	**None**

Thank you for asking me to review the authors' responses to my original comments. I am happy that they authors have responded appropriately to my comments and have made changes that strengthen and clarify their paper.

I do still feel that the paper would be easier to read quotes could be embedded in the text, rather than having to flick back and forth between the text and tables. I note the authors are unable to do this due to the word limit.

I would like to suggest that BMJ Open considers allowing an extended word count for qualitative, as some other journals do. I believe this is both methodologically and epistemologically appropriate for qualitative research given that the 'data' in quali research is text and thus inevitably takes up a greater proportion of the word count.

**Reviewer**	**3**
**Name**	**Young, Dana**
**Affiliation**	**University of Melbourne, Jack Brockhoff Child Health and Wellbeing Program, Centre for Health Equity, Melbourne School of Population and Global Health**
**Date**	**10-Sep-2024**
**COI**	**none**

This is an important study and your results have been presented clearly and succinctly A very minor comment, to strengthen your discussion I would suggest a little bit more framing against the literature. I think it would be helpful to point to research which has looked at the recovery experiences of children and families with a disability post-disaster (Peek & Stough from the USA for example), this field is very small and your research can contribute here.

I can also point you to our research which has explored the need for disability services to explicitly support the mental health of parents - and how staff are often not confident to address this, even when working in a family-centred environment. Your research again helps validate the importance of supporting parental health in order to support the child, and how when loss of services occur the implications can be detrimental to the family supporting the child. https://onlinelibrary.wiley.com/doi/abs/10.1111/hsc.13350

https://www.tandfonline.com/doi/full/10.1080/09638288.2022.2117865

VERSION 2 - AUTHOR RESPONSE

Reviewer: 1
Dr. Rebecca Langford, University of Bristol
Comments to the Author:
Thank you for asking me to review the authors' responses to my original comments. I am happy that they authors have responded appropriately to my comments and have made changes that strengthen and clarify their paper.

I do still feel that the paper would be easier to read quotes could be embedded in the text, rather than having to flick back and forth between the text and tables. I note the authors are unable to do this due to the word limit.

I would like to suggest that BMJ Open considers allowing an extended word count for qualitative, as some other journals do. I believe this is both methodologically and epistemologically appropriate for qualitative research given that the 'data' in quali research is text and thus inevitably takes up a greater proportion of the word count.

Thank you. We have now added the quotations to the main text following the editors advice on the word count.

Reviewer: 3
Ms. Dana Young, University of Melbourne
Comments to the Author:
This is an important study and your results have been presented clearly and succinctly A very minor comment, to strengthen your discussion I would suggest a little bit more framing against the literature. I think it would be helpful to point to research which has looked at the recovery experiences of children and families with a disability post-disaster (Peek & Stough from the USA for example), this field is very small and your research can contribute here.
I can also point you to our research which has explored the need for disability services to explicitly support the mental health of parents - and how staff are often not confident to address this, even when working in a family-centred environment. Your research again helps validate the importance of supporting parental health in order to support the child, and how when loss of services occur the implications can be detrimental to the family supporting the child.
https://onlinelibrary.wiley.com/doi/abs/10.1111/hsc.13350
https://www.tandfonline.com/doi/full/10.1080/09638288.2022.2117865

Thank you for bringing your work to our attention. We have added a further sentence to the end of paragraph four in the discussion.
"Health professionals may lack confidence and skills to support the needs of parental mental health and wellbeing within their professional roles, and there are evaluations of tailored training for key workers and managers with encouraging results." [40]

Kind regards,
Dr Hannah Merrick
On behalf of all authors.
